# Liver abscess in the caudate lobe caused by a fishbone and treated by laparoscopy: a case report

**DOI:** 10.1186/s12893-021-01457-z

**Published:** 2022-01-08

**Authors:** Feng Xia, Peng Zhu, Xiao-ping Chen, Bi-xiang Zhang, Ming-yu Zhang

**Affiliations:** 1 Department of Hepatic Surgery, Hepatic Surgery Center, Institute of HBP Surgery, Tongji Hospital of Tongji Medical College of Huazhong University of Science and Technology, 1095, Jiefang Avenue, Wuhan, Hubei China; 2 Department of Digestive Medical, Tongji Hospital of Tongji Medical College in Huazhong University of Science and Techology, Wuhan, Hubei China

**Keywords:** Caudate lobe, Liver abscess, Fishbone, Perforation

## Abstract

**Background:**

Ingestion of fish bones leading to gastric perforation and inducing abscess formation in the caudate lobe of the liver is very rare.

**Case presentation:**

A 67-year-old man presented to our hospital with a 2-day history of subxiphoid pain. There were no specific symptoms other than pain. Laboratory tests showed only an increase in the number and percentage of neutrophils. Contrast-enhanced Computerized tomography (CT) of the abdomen showed two linear dense opacities in the gastric cardia, one of which penetrated the stomach and was adjacent to the caudate lobe of the liver, with inflammatory changes in the caudate lobe. We finally diagnosed his condition as a caudate lobe abscess secondary to intestinal perforation caused by a fishbone based on the history and imaging findings. The patient underwent 3D laparoscopic partial caudate lobectomy, incision and drainage of the liver abscess, and fishbone removal. The procedure was successful and we removed the fishbone from the liver. The patient was discharged on the 9th postoperative day without other complications.

**Conclusions:**

Liver abscess caused by foreign bodies requires multidisciplinary treatment. Especially when located in the caudate lobe, we must detect and remove the cause of the abscess as early as possible. Foreign bodies that perforate the gastrointestinal tract can penetrate to the liver and cause abscess formation, as in this case. When exploring the etiology of liver abscesses, we should investigate the general condition, including the whole gastrointestinal tract.

**Supplementary Information:**

The online version contains supplementary material available at 10.1186/s12893-021-01457-z.

## Background

The presence of foreign materials on or in the liver is uncommon [1, 2]. In general, most ingested foreign bodies pass smoothly through the gastrointestinal (GI) tract. Less than 1% of patients with intestinal perforation have perforation due to foreign bodies. Endoscopy may be helpful if performed before foreign body migration and mucosal healing. US and CT may help in the diagnosis and treatment planning of these unusual foreign bodies. In China, many foreign bodies may cause severe perforation, including toothpicks, fish bones, dates, and chicken bones. As in our case, complicated hepatic foreign bodies should be removed by laparoscopy or laparotomy after diagnosis. The ingestion of fishbones can cause perforations in the following situations: (1) the swallowed fish bones are too sharp; (2) the peristaltic speed of the lesser curvature is fast; (3) the wall of the lesser curvature is relatively thin; and (4) digestive tract dysfunction [3–7].

Liver abscesses have many causes, but fishbone perforation is a rare cause. In addition, fish bone insertion into the caudate lobe of the liver is even rarer. Because the caudate lobe is close to vital vascular structures, surgical resection is difficult and risky. It is considered the last area of laparoscopic liver surgery and was considered a difficult area for surgery in the past. Therefore, surgical treatment of liver abscess caused by a fishbone penetrating the caudate lobe of the liver is also a great challenge [8–10]. In recent years, with the development of liver anatomy and advances in diagnostic techniques, reports of caudate lobectomy are increasing.

So, here we report a case of an abscess in the caudate lobe of the liver secondary to gastric perforation caused by a fishbone.

## Case presentation

**Fig. 1:**
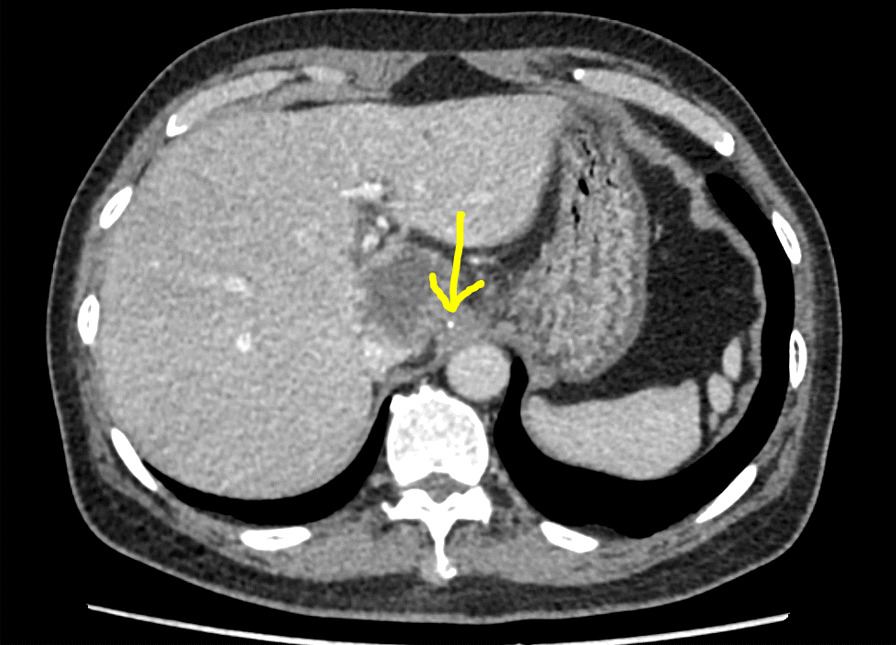
Enhanced computed tomography (CT) revealed an abscess in the caudate lobe of the liver and a needle-like foreign body near the cardia

**Fig. 2:**
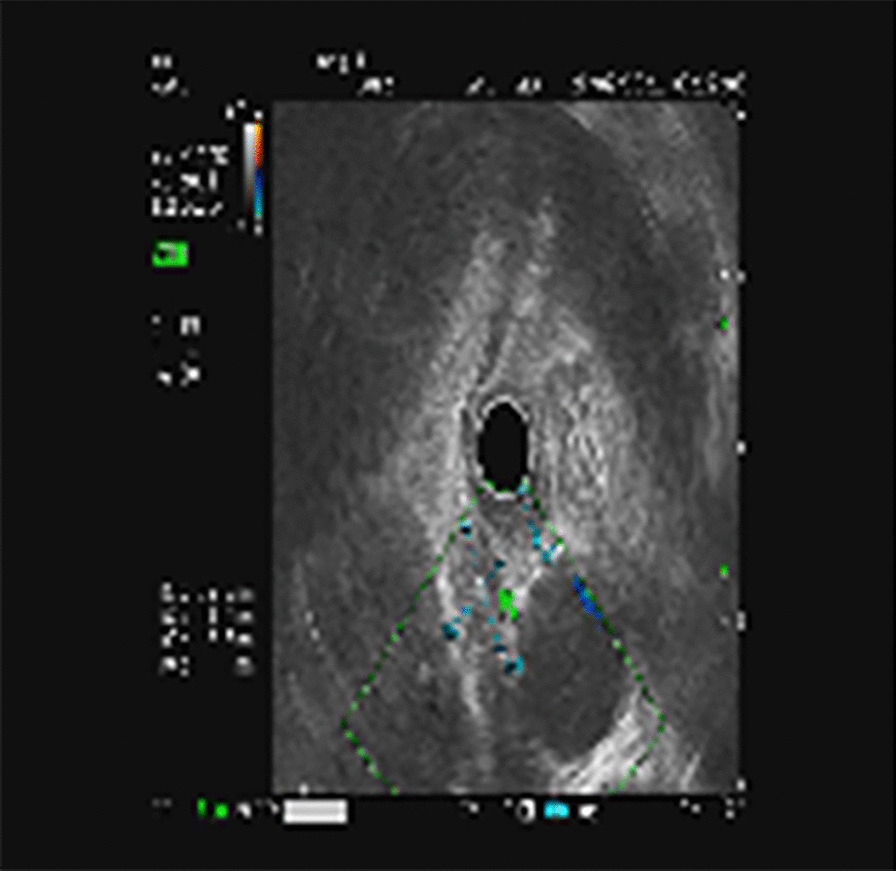
Endoscopic ultrasonography: mixed echo lesions in the hepatogastric space: combined with the history, foreign body wrapping may occur

**Fig. 3:**
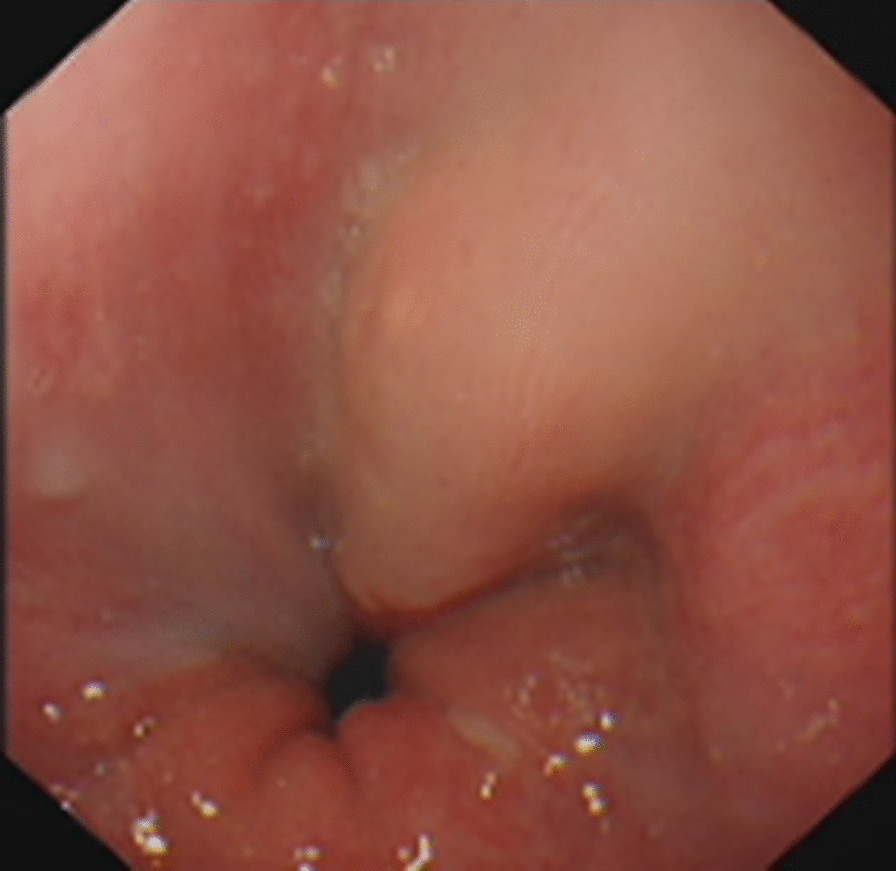
About 1 cm × 1 cm from the incisor, there is a cord-like protrusion

A 67-year-old man was referred to the general practice department of our hospital due to subxiphoid pain. The patient was emergently admitted to our hospital. He had a 7-day history of eating fish. Before emergency admission, no special treatment was performed, except for an abdominal CT at the local hospital. He had a medical history of penicillin anaphylaxis. On emergency admission, his body temperature was normal. On the evening of admission, relevant laboratory investigations, such as routine blood tests, liver and kidney function, electrolytes, and inflammatory proteins were performed. Upon physical examination, the patient’s right upper quadrant was mildly tender to palpation without rebound tenderness. The white blood cell count was 18,150/μL (91.1% neutrophils). The C-reactive protein level was 125 mg/L. Serum bilirubin was normal. Glutamic oxaloacetic transaminase, and glutamic-pyruvic transaminase levels were 42 U/L, and 65 U/L respectively. On the first day after admission, considering that the patient had elevated inflammatory proteins and leukocytes, we performed a chest CT to see if there was a lung infection. Chest CT was also done as a routine test because of the Covid-19 pandemic. Chest CT revealed an infectious lesion in the upper lobe of the left lung with segmental atelectasis. On the second day after admission, we decided to perform a contrast-enhanced abdominal CT to better understand the patient’s abdominal cavity situation. The contrast-enhanced CT revealed two linear dense shadows in the gastric cardia, one of which penetrated through the gastric wall and was adjacent to the caudate lobe of the liver, accompanied by inflammatory changes in the caudate lobe. A patchy low-density shadow with enhanced edges measuring 41 mm × 38 mm was observed (Fig. 1). To further understand and clarify the depth and localization of the caudate lobe mass, we decided to perform endoscopic ultrasonography (EUS) examination under sedation. The EUS under sedation showed mixed echo lesions in the hepatogastric space (Fig. 2). Combined with the patient’s history, there was a possibility of foreign body wrapping, however, the EUS was unable to detect any lesions in the gastric wall (Fig. 3). Blood culture results did not detect bacteria 2 days after admission. The results of the examination interfered with our judgment.

On the third day after admission, we also performed upper gastrointestinal radiography (iodinated water) examination to further confirm the presence of foreign material on the gastrointestinal tract, but the results did not reveal obvious leakage of contrast medium in the esophagus and stomach.

Based on the patient’s history, signs and symptoms, and auxiliary examination results, we made a definite diagnosis of liver abscess caused by a fishbone piercing through the gastric wall and penetrating the caudate lobe.

**Fig. 4:**
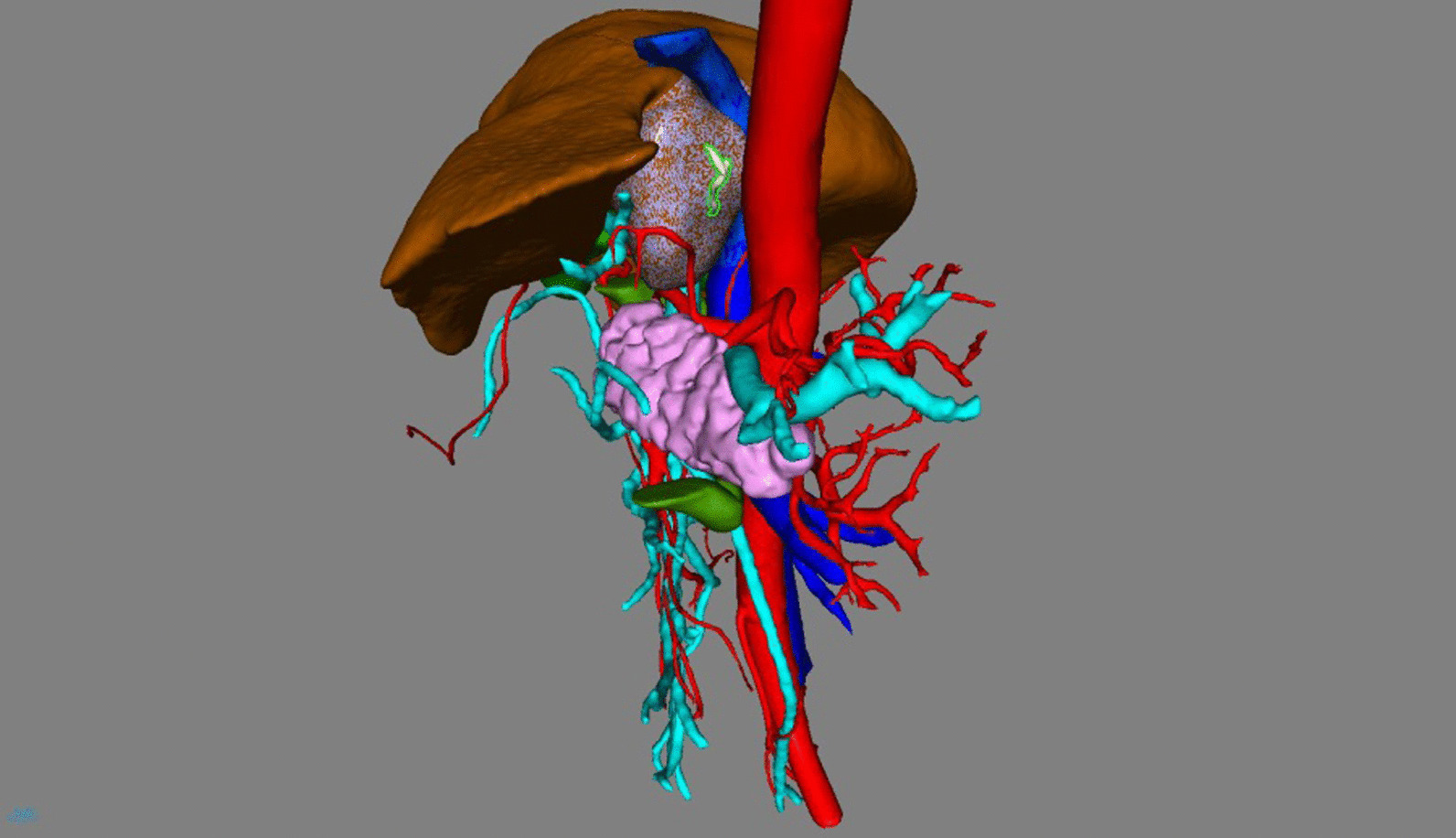
In this model, red represents arteries, blue represents veins and white represents the fishbone. From this model, we can realize the location of the fishbone and its adjacency

On day 4 of hospitalization, we performed a model reconstruction of the surrounding liver before surgery to better evaluate the general situation (Fig. 4).

On the 5th day after hospitalization, we decided to perform a 3D laparoscopic partial resection of the caudate lobe of the liver, incision and drainage of the liver abscess, and repair of the gastric perforation.

**Fig. 5:**
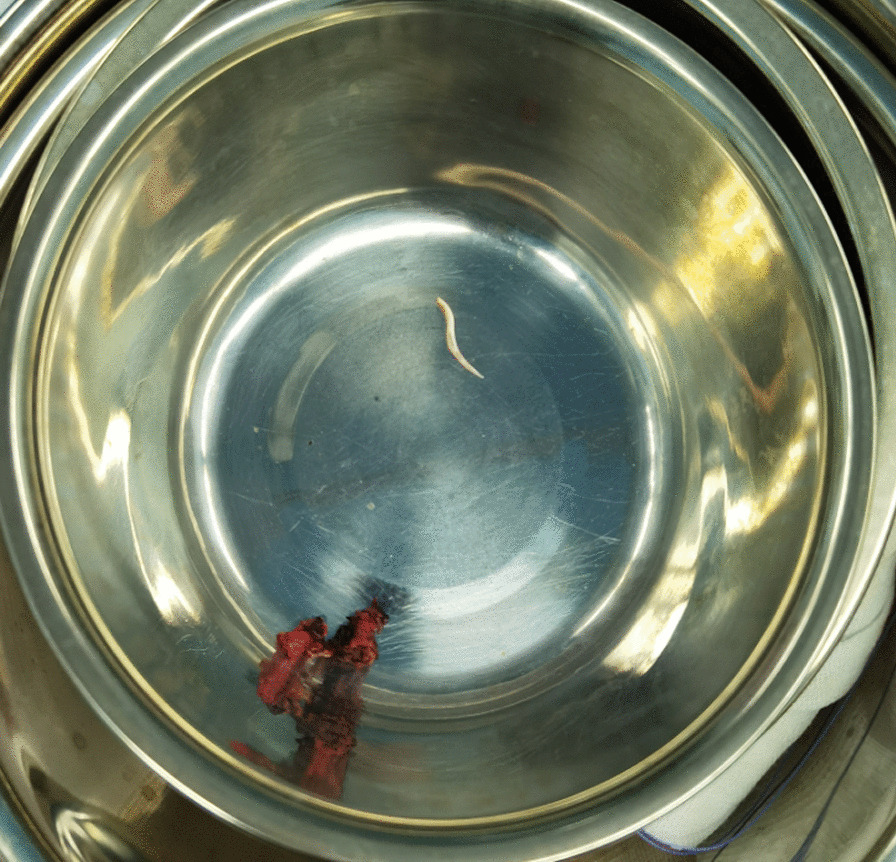
There was an S-shaped fishbone that was taken out from the caudate lobe of the liver

Laparoscopic exploration revealed no significant ascites in the abdominal cavity, the liver was soft in consistency, and no significant sclerosis or tumor lesions were found. After exposing the caudate lobe, a sigmoidal fishbone could be seen outside the caudate lobe, and it was connected to the lesser curvature of the stomach (Fig. 5).

The liver surface was covered with white pus. Inflammation, edema, and adhesions were observed in the lesser curvature of the stomach penetrated by fish bones, and perforation was observed. No other abnormalities were observed. Liver abscess, foreign body, and gastric perforation were diagnosed.

During the procedure, firstly, we cut the ligamentum teres, the falciform ligament, the left part of the coronary ligament, and the left triangular ligament. Then we turned the left lateral lobe of the liver to the right, incised the lesser omentum close to the surface of the liver, exposed the caudate lobe, and pulled out the fishbone under direct laparoscopic vision. We also removed parenchyma with a radius of 1.5 cm around the fishbone until the abscess cavity was exposed. The pus in the abscess cavity was aspirated, and part of it was sent for bacteriological examination; at the same time, the lesser omentum around the caudate lobe, close to the lesser omentum at the lesser curvature and lower esophageal-cardia was resected posteriorly up to the anterior abdominal aorta, and repeated exploration along the lower esophageal-cardia revealed no leakage. After a complete examination of the condition in the abdominal cavity, the specimen was intactly removed. The first porta hepatis and inferior hepatic vein were not blocked during the operation. Postoperative histopathological examination revealed a large number of neutrophils infiltrated into some areas of liver tissue, along with necrosis, which was consistent with the changes observed in liver abscesses. The patient underwent iodine hydrography on the 4th day after surgery, and the examination results showed no significant contrast agent leakage in the esophagus and stomach. The patient had an uneventful postoperative recovery and was discharged on the 9th postoperative day (Additional file 1).

## Discussion and conclusions

Inadvertent ingestion of fish bones is very common in China, especially in the elderly, children, and people with mental disorders. At the same time, people with diminished oral sensation are also prone to such conditions. The sharp edge of fish bones easily scratches the gastrointestinal mucosa and even causes peptic ulcers and perforation in severe cases. Although most of the ingested foreign bodies are excreted through the gastrointestinal tract within a week without other complications, unfortunately, gastrointestinal perforation occurs in less than 1% of patients [11]. In this case, the patient will have acute symptoms and should promptly visit the hospital. Such patients may have symptoms similar to other inflammatory reactions, or chronic fever due to a liver abscess caused by a fishbone. Currently, early diagnosis of liver abscess caused by fishbone perforation is difficult and should be aided with imaging and patient self-reports. CT and endoscopies are the clinically determined imaging methods. At the same time, selecting the appropriate timing for surgery is also a good means of diagnosis and treatment [12–14]. In recent years, studies have shown that CT has a high sensitivity to fishbones and other foreign bodies, some studies have shown that the diagnostic sensitivity of CT for perforation caused by fishbone is as high as 70%, even as high as 100% in some studies [15–17].

Although our patient had no fever or nausea and vomiting, we diagnosed the patient’s condition as a liver abscess caused by the perforation of a fishbone into the caudate lobe based on the patient's self-report and enhanced CT findings.

Endoscopy may be helpful if performed before foreign body migration and mucosal healing. Regarding the success rate of endoscopy, Jason Saltiel et al. [18] performed endoscopies on all patients with ingested foreign bodies, and only 78% found foreign bodies. They concluded that foreign bodies visible on X-rays are easier to see in endoscopies, and factors such as age, X-ray visibility, and type of suspected foreign body may affect the possibility of foreign bodies being seen and removed endoscopically. For oral contrast examination of the digestive tract, Jae-Kwan Jun et al. [19] believed that eliminating the use of oral contrast agents in abdominal pain examination could help achieve more timely care. Similar to the opinion of Jun et al., Kessner et al. [20] believed that for most patients with non-traumatic acute abdominal pain, oral contrast examination did not contribute to the diagnosis.

So, we believe that oral contrast agents should be used as little as possible in the later diagnosis of non-traumatic abdominal pain. The perforation might have been difficult to detect using gastroscope under sedation and upper gastrointestinal radiography with iodinated water in our case. However, combined with the patient's chief complaint, enhanced CT, endoscopic ultrasonography, we were able to arrive at the diagnosis.

It is worth noting that we do not use MRI to diagnose patients during the treatment process, but we believe that MRI can also improve the ability to diagnose accurately. In a case report of a hepatic abscess secondary to a rosemary twig migrating from the stomach by Karamarkovic et al. [21], MRI was used during the diagnosis process to further determine the presence of a liver abscess. They accurately determined the location of the liver abscess, providing value for the implementation of surgery.

During laparoscopic exploration, a fishbone perforating the caudate lobe of the liver was observed under direct vision, and the omentum was wrapped near the caudate lobe. The lesser omentum was incised close to the surface of the liver, the caudate lobe was exposed with dissecting forceps, the fishbone penetrating the liver was pulled out under direct vision, liver parenchyma with a radius of 1.5 cm was removed until the abscess cavity was exposed. The pus was aspirated with an aspirator, some of it was sent for bacteriological examination, and a drainage tube was placed beside the abscess cavity. The abscess was flushed and drained, and the trocar hole was closed.

During the operation, we should pay attention to the partial resection of the caudate lobe, the particularity of its location, and the complexity of its anatomical structure. The caudate lobe is surrounded by the three portae of the liver, it is adjacent to the inferior vena cava, portal vein, hepatic vein, and bile duct. The operating space is narrow, exposure is very difficult, intraoperative bleeding is difficult to control, and the operation risk is high. Advanced diagnostic techniques, familiarity with the anatomical characteristics of the caudate lobe, selection of the best surgical method and surgical approach, blood flow occlusion techniques, and prevention of postoperative complications are the basic requirements to guarantee a successful operation and prolonged survival [22–26].

We use hepatectomy as the main treatment for patients, but there are other methods to treat foreign body-induced liver abscesses. For example, Horii et al. [27] treated it by first removing the foreign body by liver puncture and draining the abscess, followed by antibiotic treatment, and a good therapeutic effect was obtained. So in future treatment, we believe that percutaneous drainage by an interventional radiologist can be part of the treatment options.

*Klebsiella pneumoniae* is the most common pathogen that causes liver abscesses. However, in our case, no bacteria were found at the time of blood culture, but a culture of the purulent fluid from the liver abscess removed intraoperatively revealed growth of *Streptococcus intermedius*. This is the same genus that constitutes part of the normal bacterial flora of the human mouth, nasopharynx, and GI tract. We, therefore, suspect that oral, pharyngeal and GI streptococci have the potential to cause liver abscesses. So, the detection of these bacteria can also provide evidence that the liver abscess has developed from a foreign body that has penetrated the gastrointestinal wall to the liver.

In this case, no foreign body was found by the gastroscope under sedation, which may be related to the small size of the foreign body. Compared with B-ultrasound and X-ray, CT or enhanced CT is a more sensitive method for diagnosing foreign bodies in the digestive tract, especially in coronal or sagittal reconstruction, with high density and spatial resolution. CT can not only find all foreign bodies, but it can also mark the position, shape, and adjacent relationship with the surrounding organs. Combined with the results of this endoscopic examination, it can be used to judge the operation track of foreign bodies in vivo, and it is an important reference for surgeons when formulating the surgical plan. However, there is a certain rate of missed diagnosis in imaging examination, and a negative result can not rule out the diagnosis of a foreign body, so we need to make a comprehensive judgment combined with the clinical findings.

In conclusion, when a patient with a relatively special liver abscess is found, we need to listen carefully to the patient's chief complaints and ask carefully ask about the history. When upper GI endoscopy suggests the possibility of a perforation in the gastrointestinal tract, we need to be vigilant about infection of the abdominal organs, and enhanced CT scans should be performed to provide a sufficient basis for the treatment plan.

## Supplementary Information

**Additional file 1:** Chronology for diagnostic process.

## Data Availability

The datasets used and analyzed during the current study are available from the corresponding author on reasonable request.

## Abbreviations

MRI: Magnetic resonance imaging
CT: Computed tomography
GI: Gastrointestinal
US: Ultrasound
