# Supplementary figures and images for: Liver abscess in the caudate lobe caused by a fishbone and treated by laparoscopy: a case report
Source: BMC Surg. 2022 Jan 8;22:6. doi: 10.1186/s12893-021-01457-z (PMC8741587; doi:10.1186/s12893-021-01457-z)

## Slide 1
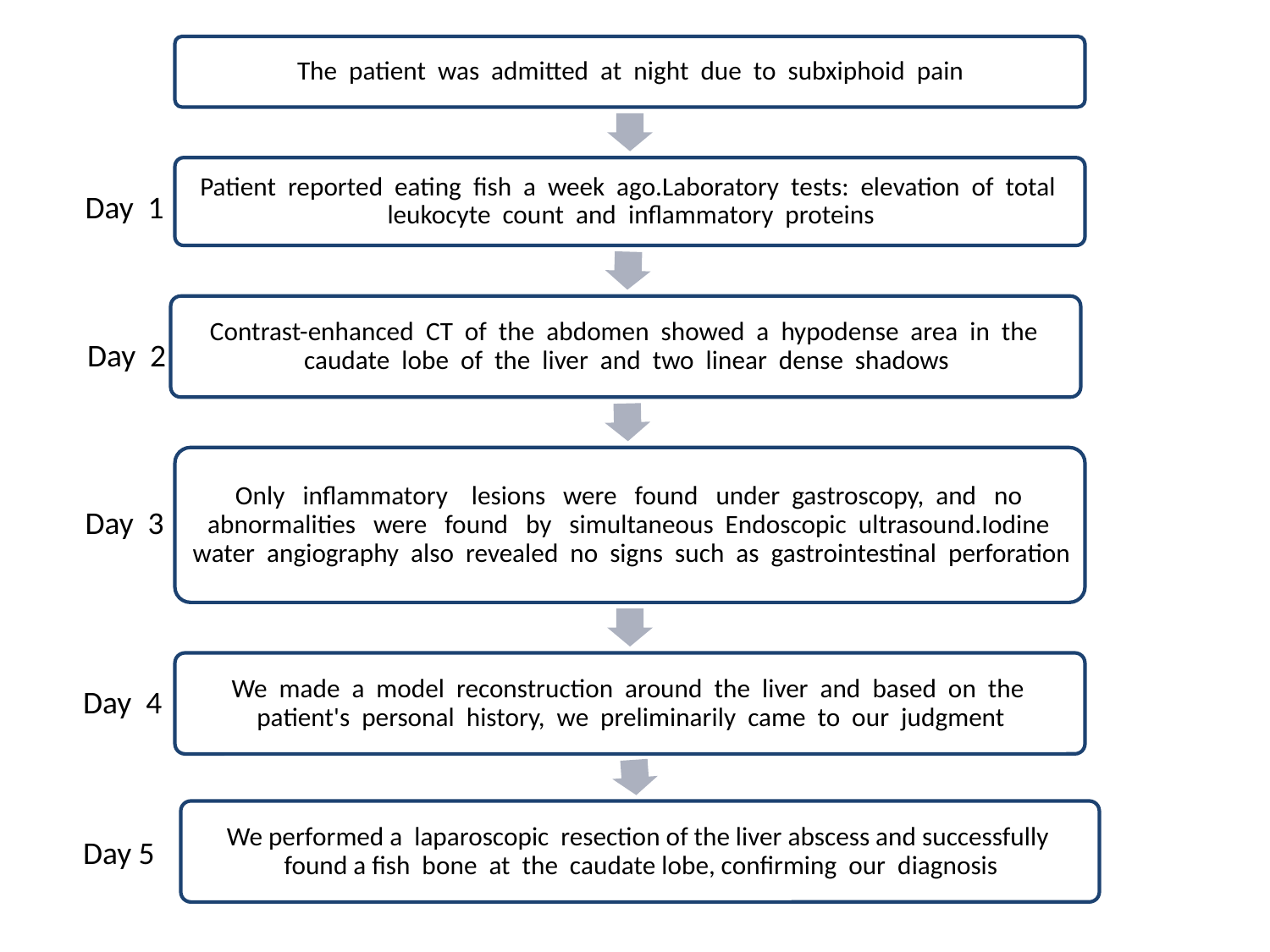

Day 1
Day 2
Day 3
Day 4
Day 5

Supplement: Supplementary file 1 — Additional file 1: Chronology for diagnostic process. [file 12893_2021_1457_MOESM1_ESM.pptx]
